# Continuous Gum Work

**Published:** 1858-12

**Authors:** W. B. Roberts


### CONTINUOUS GUM WORK.

BY DR. W. B. ROBERTS.

TO THE EDITORS OF THE REGISTER.—*Gentlemen*:—By your last number I perceive that you hold me to the promise I rashly made at the 4th Annual American Dental Convention, of furnishing an account of the method of making continuous gum work. The task I find is a more formidable one than I anticipated, and I regret it has not fallen to one more familiar with the use of the pen, and more competant than myself to do justice to a subject of so much importance. Why it has not before this been fully treated by those who have written so ably upon other branches of mechanical dentistry, it is difficult to say, unless it be that a knowledge of the art is still limited to a few, or that *its being patented* stands in the way of a favorable notice. However that may be, the admitted beauty and durability of the work, and its being generally adopted by the best practitioners, render a knowledge of the method of making it desirable. I shall attempt but a plain practical account of it, such as one may be supposed to offer to an actual pupil entering upon its study, after having made himself conversant with the usual practice in preparing and fitting gold or other metallic plates.

We will suppose the work before us is the preparation of two upper sets. In following out the different processes, a few remarks may be introduced, suggesting methods of avoiding difficulties most likely to be met with.

IMPRESSIONS.—When the plaster, mixed in the usual way, is nearly ready to set, you should fill the cup, raising the center a little higher than the edges. Introduce it gently into the mouth of the patient, after placing a napkin under the chin, and then carefully work and press the cup up to the roof of the mouth. The head of the patient should be inclined forward, so that the saliva may flow out upon the napkin. By neglect of these precautions I have known patients in the hands of inexperienced operators to suffer severely from choking. The cup should be held firmly in its place, not allowing it to be disturbed in the slightest degree, until it is ready to be taken out. The process is to be repeated for the two above-mentioned cases. Gutta percha in partial cases may be used to great advantage for taking impressions.

PLATES.—Leaving each one to his own method of making plaster casts, the next process is the preparation of the dies and plates, and this presents some peculiarities which distinguish it from the routine pursued in making gold plates. In every piece of platinum work the edge of the plate should be turned over, in order to give a smooth finish to the edge, and under which the gum and body flows, adding much strength to the entire piece. The body and gum, when laid over the surface of the plate, should not quite reach the posterior edge, but about a quarter of an inch of the metal should be left exposed; the rest of the plate is to be covered with the gum work, which should slope off smoothly to the platinum, so as not to present any roughness to the tongue, and the surface of it is to be polished, to imitate the appearance of the natural rugæ of the palatial arch. The sloping edge may be obtained by soldering a three-sided wire across the plate, near to its edge; or by a method which I find preferable for its greater neatness, and the increased stiffness it gives to the plate. This is in building upon the plaster model (taken from the impression) a little triangular ridge in the same position the wire would occupy, extending in crescent form from the back edge of one wisdom-tooth to that of the other—the curve being forward. The ridge should slope very gradually toward the back edge of the model, and the rise, when transferred to the plate, will hardly be perceptible to the tongue. The front side of the ridge serves as a shoulder for building the gum work against. I have sometimes carried the gum out to the edge of the plate; but this requires a very accurate fit. This result may also be obtained nicely by doubling the posterior edge about one-fourth of an inch in width, and finishing against the edge of the piece put across. We will suppose the first two methods (the wire and the triangular ridge) to have been adopted for obtaining the ridge upon the plates, one for each of the two upper sets. The mouth, when the impression is taken, should be carefully examined, to note the position of the muscles, and to determine the proper depth of the plate. You are then to take a little beeswax or plaster, and build a rim around the edges of the plaster models as high as you wish the plates to come, thus forming a shoulder, which, when the two parts of the die are closed over the plate, will form an angle of about (50) fifty degrees, which may be bent down, as will be hereafter described. The dies may be prepared of zinc or other metal, or any alloy you are accustomed to employ for this purpose—some precautions are to be taken in striking the plates, which are not required in swaging those of gold or other materials that are not to be afterward exposed to high temperature. Being cut by the pattern leaving it to project about one-eight or one-sixteenth of an inch upon the shoulder, for a rim, the plate is to be laid upon the metallic die and gradually worked down with a wooden or horn hammer to a tolerably close fit, before attempting to swage it. It may be split in front if necessary, and also at the heels, and afterward restored by soldering the same as is practiced with gold plates; but this is better omitted in cases that can be swaged whole. Great care is required to avoid bursting or tearing the platinum in the swaging. Annealing should be often repeated; and previous to each annealing, the plate should be cleaned with nitric acid in order to remove all particles of zinc and foreign substances that may be taken up and adhere to the platinum. These, if allowed to remain, would be incorporated with the platinum by the action of the heat in annealing and baking, which would seriously injure it. The swaging should be continued with hard blows, with a heavy hammer or drop, forcing the platinum into every depression in the dies; and the greatest care should be taken in completing the work, that the sur face is smoothly finished, showing no dents or scratches. We will suppose the two plates we have commenced to come out in different conditions—the one we intend to put the wire across to finish the gum work, is disfigured upon the surface with dents and imperfections, resulting from incautious swaging, and with a hole in it, caused by being annealed without thorough cleansing from the particles of zinc or other metals it came in contact with, which made it extremely brittle. Zinc or lead is particularly destructive to platinum when the metals are heated together. The other plate, we suppose, is in good order, with the exception of the splits purposely made before swaging, and which are now to be closed up by soldering with pure gold. The hole in the other is also to be mended by introducing over it, on the palatial side of the plate, a piece of platinum, and soldering this with gold. Soldering at the same time across at the point heretofore described.

ARTICULATION.—You will now proceed to fit the plate in the mouth of the patient, noticing particularly whether it rocks or loses its adherence when pressed upon one side or in front. If satisfactory, next place upon the plate a ridge of wax, which will serve to show the length and position the teeth are to occupy, and serve as a temporary fastening while arranging them. The best swaged of the two plates we will suppose now makes the best fit, and being provided with wax, is placed in the mouth of the patient, who is directed to shut the mouth in a natural way, and cause an impression of the under teeth to be made in the wax above. A vertical line is then to be drawn in the wax in front, marking the division between the two central incisors. We have now a perfect impression of this one ; but no provision for determining the proper length to give the teeth. For this the upper lip should be allowed to assume a natural, easy position, and so much of the wax should then be cut off, as to leave about one sixteenth of an inch in the place of the central incisors visible below the edge of the lip ; towards the bicuspeds the length should slightly diminish. The other plate, when furnished with the wax, we suppose is placed in the mouth of a person who has, for years, enjoyed the use of only the front teeth—when such an one is directed to close the mouth, it will be found almost impossible to make two successive impressions of the lower jaw in the wax coincide; the patient does not bite twice in the same place. Close observation and the greatest care, will, therefore, be required in determining the proper place for the teeth, in order to obtain a natural expression. The face, moreover, in this instance, is sunken, and requires filling out to restore its original contour. For this purpose wax must be added whenever it is needed to form prominences, and depressions must be moulded in it where these are natural, as for instance, directly in front, where a fullness would give the appearance of a swollen lip. For the prominences or cheek restorers, designed to fill out the sides of the face, the projection of wax must not extend further towards the front of the mouth than the line between the second bicuspid and the second molar, if brought beyond this point, they distort the features, and show; when the mouth is opened, the shape and dimensions of the prominences should be obtained by repeated trials, observing carefully the effect of every alteration, as the patient is purposely kept in good humor, and without exciting suspicion of the object, induced to laugh and give to the features their changes of expression. The dentist should watch his patient as closely as an artist his sitter, in painting a portrait from life. And, here let me say, that this, that I call *artistic dentistry*, has been, and still is, grossly neglected; for, how often do we see persons in whose faces we discover a dilapidated condition of the entire outline of the features. This can all be attributed to want of knowledge, or neglect on the part of their dentist. After having ascertained the necessary length and projection to be given to the cheeks and teeth, place the wax and plate in cold water, to render it as hard as possible (as we will suppose this to be a set to match an artificial under set) we will then place wax upon the under plate, the length as near as possible that we wish the under teeth, soften it, and placing both plates and wax in the mouth, let the patient gently close the mouth until the upper wax presses into the under sufficiently to allow the lips to close or touch in a natural and easy manner. This will show the length required for the under teeth. Let the patient open and shut the mouth, merely touching the wax together—let it be repeated several times, opening the mouth as wide as possible, and bringing the jaws suddenly together, so that there may be no mistake. When right, let the gums be pressed together so as to hold the whole mass firmly without disturbing the wax; then, with three pins or pivots of wood, fasten them together, running one near each corner of the mouth, through the edge of the under wax in the upper, the other pin into the edge of the upper, running down into the under wax at a point midway between the other two pins. Then remove the whole mass at once, place it on the articulating frame and run in the plaster. After it hardens remove the upper plate, place it upon a piece of paper, and draw a pencil mark around it, representing the projection the wax is intended to give the teeth and cheeks. Turn it over and draw another line. With these two lines or drawings as a guide, the teeth, body and gums may be placed as accurately upon he plate, as the wax had been, (which it will be necessary to remove by the process of imbedding, lining and soldering,) without fitting or trying it in the patient's mouth, during the process of making the set. Yet, it is always desirable to introduce them into the mouth once or twice, for instance, after the teeth are soldered fast—and again when the first coating of body has been applied. This prevents all possibility of mistake.

ARRANGING TEETH.—The next operation is imbedding the artificial teeth in the ridge of wax prepared to receive them. This point requires especial care, and also some artistic taste; for, a slight variation in the position of a tooth may give a decided and unfortunate change of expression to the whole set. You will begin by cutting a bed for a central incisor, on one side of the central line marked in the wax, as deep as the thickness of the tooth, and laying this in its place, where it will be held by the wax. One tooth after another is to be thus placed, till the set is complete. Now, I will take the piece in my hand, place my thumb across the teeth to represent the lip, and observe the effect. The central line is right, the lateral incisors are even; but they stand too formal and stiff. I move them up a little to make them shorter than the centrals and incline them slightly, so that one corner of each nearly touches the adjoining central incisor: this gives a more natural expression. I move the canine teeth also, to make them a little more prominent. Attention must now be given to avoid in the general arrangement a defect very common in sets of artificial teeth, viz: too great exposure of the back teeth giving the appearance when the mouth is opened, of its being filled with teeth. This comes of the curve of the piece being carried too far back, when it should be limited merely to the space occupied by the six front teeth. The bicuspids and molars must therefore be pushed in, the first bicuspid being nearly hidden behind the canine, and those beyond extending back in a straight line diverging slightly as they approach the end of the jaw. The teeth may now be covered with a thin coating of shellac, which will often prevent their becoming rough, when exposed to the heat of the furnace in soldering. They are next covered over with a coating of clean plaster, which holds them firmly while the linings are put upon them, and then imbedded in a mixture of equal parts of plaster of Paris and asbestos. When this hardens, the wax may be removed from the backs of the teeth, leaving them bare for receiving the permanent linings and attachments to the plate.

Lining and Fastening.—One set may have been furnished with the teeth known as "Robert's teeth," which are made with but one long pin. Under these you will fit a slip of tin or sheet lead, passing from one canine to the last molar on the other side, and a corresponding piece crossing this from the other canine to the other molar. These are patterns from which to cut the slips of platinum. The latter when shaped are to be punched full of holes (through which the body runs, giving increased stiffness to the whole), and placed under the pins, standing edgewise upon the alveolar ridge. Other strips may be introduced wherever you think the work requires stiffening and strengthening: the whole lining may thus be doubled or tripled. A wire also may be run across from one bicuspid to the other, which will counteract any tendency in the work to spread. The pins may now be bent down over the slips holding them in their places, until the fastenings are completed by soldering them together. The linings may thus be put in, in from ten to twenty minutes.

The set provided with the other style of teeth, having two pins, is to be managed in another way. Place the piece on the table, the back edge directed toward you, and the teeth uppermost. Cut platinum into slips one for each tooth, of width equal to that of the tooth and half the spaces beside between it and the one next to it. These, when arranged in their places, will then form a continuous lining behind all the teeth. Bend up about one-eighth of an inch of one end of each strip at an angle of forty-five degrees, and divide this portion by slitting it with four or five cuts. Touch the points of the pins with a camels hair brush dipped in rouge or other easily transferred coloring-matter, and apply the slips of platinum, one after the other, to their respective teeth, with the split end resting on the plate, that they may receive the mark for the holes for the pins. Then punch the holes. The upper ends may now be trimmed off nearly down to the holes; and the projecting sides be slit evenly with the edges of the tooth from the top about halfway down, leaving the narrow slips attached. With a small pair of tweezers place the lining on each tooth, letting the pins come through the holes punched for them, and then pinch each pair of pins together with pliers, thus fastening the linings firmly to the teeth. The slips of platinum at the edges are then to be interlocked with the slips belonging to the adjoining teeth till all are bound together in a continuous lining. The bottoms or split ends resting on the plate should then be pressed down so as to come in close contact with the plate, this being necessary to insure adhesion by soldering. With a sharp pointed instrument make a scratch along the plate about one-sixteenth of an inch from tee foot of the linings, which will prevent the solder flowing out upon the plate. Place a small bit of pure gold upon the pins, another bit at the interlocking of each pair of slips, and one or two at the base of the lining of each tooth, adding with each bit as much borax as will adhere to the little piece of solder, and no more. The piece is now ready for the next process of—

SOLDERING.—You may now gradually heat up the work in the muffle of the furnace, and when it is of a deep red, remove it on a piece of charcoal or other suitable support and solder the parts with a hydraulic blowpipe. But if this or no other one equally powerful be at hand, the piece must be left in the furnace, and a careful watch kept to remove it as soon as the solder all flows; for, exposure to high heat is apt to etch or roughen the surface of the teeth, by the adhesion of plaster and asbestos to the enamel.

After taking the work from the furnace and cooling, the next operation is to remove the plaster; and the portion of this forming the base, on the part that corresponds to the jaw, should be reserved to be used as a support for the piece in the subsequent bakings, which prevents, in a degree, change of shape or springing of the plate. Should the piece soldered in the furnace be found on removing the plaster and asbestos, etched or rough, we will restore it as described under the head of enameling or gumming. The pieces are to be worked, then placed in diluted nitric acid for the purpose of removing the adhering borax and other foreign substances, and then washed again. They are next laid upon the metallic casts to be there held while the plate is scratched or etched with a sharp point over all the surface intended to be covered with the body and gum.

FIRST COATING OF BODY.—The material we prefer over all others is Allen's preparation, called "body," as prepared by E. & L. Roberts. This being made into a thick paste, with a little water, is taken up with suitably shaped knives or spatulas, and introduced between and around the teeth, and over the palatial arch as far back as the projecting bead or the wire left near the back edge of the plate. The paste should be carefully worked and pressed into the interstices it is designed to fill, packing it solidly, and building up or keeping back according as prominences or depressions are to be produced. Then with your little knives carve out between the teeth, leaving a furrow which will produce a prominence over the fangs, resembling the roots of the teeth as they appear in the natural gums. The crowns of the teeth should be kept carefully free from the coating, as also the spaces between the teeth, lest these should be afterward baked together. Sufficient width must also be cut under along the projecting edge of the plate to admit of its being turned over, as hereafter described.

BAKING.—When the plate and teeth are thoroughly cleaned with a brush, and all loose particles of the paste removed, it is laid upon the base reserved for the purpose, and this upon a baked fire-clay tile or slide, and is introduced into the outer part of the upper muffle of the furnace, as it becomes hot it is gradually pushed farther in. When quite red hot, it is transferred to the lower muffle, and there brought to a full white heat, at which it may remain from five to twenty minutes, or until it is seen, on drawing it to the outer end of the muffle, to have a glossy appearance. It is then removed and placed in another muffle not connected with the furnace, which should be closed up while the plate gradually cools. The process is somewhat varied in the case of the set with single pins to the teeth, as these require some additional support to counteract any tendency to turn upon the pin, or otherwise move from their place. This additional protection is obtained by turning the plate with the teeth down and burying these for halt the length of the crowns in a paste of pure pulverized silex placed to receive them. This will fill the spaces between the crowns of the teeth and may be removed without difficulty after baking. Or, placing the set upon the plaster base as before, we will brace the teeth requiring support by introducing small pieces of platinum in the spaces. This precaution it may be well to adopt in any case.

When the piece is quite cool, it must be placed upon the metallic die to detect any alteration that may have occurred in its fit, and to restore it to its former shape; which may be done by pressing it down with the hand or by tapping the molar teeth with a wooden or horn hammer, and pressing the plate at the same time firmly down to the die. It may sometimes be sprung back by applying the thumbs to the air chamber and the fingers to the cutting edges of the molar teeth and pressing; or a gentle tap with the wooden hammer upon the air chamber may answer the purpose; but the safest plan is to use the die. Should cracks be produced in the body, no harm is done, as these are made to disappear in the second baking. The edge may be turned over by means of the burnisher, using also a little hammer and pliers too, if necessary. After this is done, the plate should be fitted upon the die, to be sure that the fit has not been disturbed.

It is well also to test it in the mouth of the patient before proceeding further, as any alterations required can be conveniently made in this stage; but if the work has been well done so far, it is not essential to try the fit in the mouth.

SECOND COATING.—The paste for the second coating is made the same as that for the first. It is carefully laid upon all the parts that require to be built out, and is introduced into all the crevices into which it is made to settle, by lightly tapping the piece. With the little spatula the surface is nicely carved and shaped around the roots of the teeth, as in preparing for the first baking. A little ridge is raised with the camel's hair pencil from the line between the two central incisors running back along the palatial surface to the middle of the posterior edge of the plate; and this ridge is next halved by a central line made along its whole length, by the brush or the edge of the spatula. On each side of the ridge, little prominences are to be raised to imitate the ruga of the mouth; the dentist must, after observing different mouths, exercise his taste and judgment in producing a natural effect. The second baking may then be conducted like the first, (with the exception of its being a little harder), and the subsequent cooling also; when the piece should be placed again upon the metallic die to prove its shape unaltered, in which case it is ready for the coloring.

ENAMELLING, OR GUMMING.—The "gum," being mixed as was the body, should be evenly laid on, in proportions only to be learned by experience; and in the selection of the shades of color you must be governed by your own taste, as some dentists prefer brighter and some paler tints for their work. I should recommend, however, the use of very thin coating at first; for this can be added to, and a deeper shade given by a second heat if desirable. If the teeth have been etched in soldering, a little coating of etching enamel, of the consistency of cream, should be laid upon them with a camel's hair pencil. The plate being well cleaned is then ready for the furnace; but more care is now required not to expose it too suddenly to a high degree of heat. If pushed too fast into the muffle, pieces are likely to scale off, leaving defective spots, such that it would be necessary to remove the work, cool it down, and repair with more gum. When well heated in the upper muffle, the piece may be removed to the lower one, and exposed to a full white heat from ten to twenty minutes, or until the gum has thoroughly flowed and shines like melted glass.

It is now to be removed and cooled down as before, in a cold muffle. When at blood heat take it out, and place it in water of about the same temperature, avoiding touching it with the fingers. This is an effectual method of preventing the checking or crazing of the gum, which frequently occurred in the old way of annealing, when the piece was cooled and taken in the hands. I discovered it some time ago, and communicated it to several dentists in this city; and I notice publicity has been given to it, without my receiving the credit to which I am entitled. Though it appears a simple thing, it is certainly an important improvement. At first, I placed the piece in my mouth on taking it from the muffle; and afterward I substituted warm water. The next and last operation is polishing, in which there is nothing requiring peculiar directions,—I remove from the plate any little particles of gum or body, and rub it thoroughly with fine sand-paper, then with a fine stick and pumice stone or fine emery, and lastly with a steel burnisher or bloodstone, with a little soap suds.

CONTINUOUS GUM APPLIED TO PARTIAL SETS.—Partial cases may be made of continuous gum; but the work is so various in its nature, that the dentist must necessarily depend much upon his own judgment. Difficult cases will constantly present themselves, that will require the exercise of much study and ingenuity, in which the general instruction that can be given in words, may be of but little service. The first attempt of this kind in my own experience, was in replacing two central incisors. Taking two continuous gum teeth, I placed upon them a platinum lining, slitting this down along the edge of one tooth nearly through the piece and up the edge of the other tooth by a parallel cut, leaving the two parts joined together by a narrow slip. This allowed sufficient motion between the teeth, so that they could be adjusted as desired. I then placed a bit of tissue paper on the plaster model, covering the spot to be occupied by the teeth and gum, to prevent the adhesion of the body to the plaster, and holding the two incisors in their places, I worked the body into all the depressions of the gum and around the roots of the teeth. I then removed the whole from the model, and placed the piece in a paste of pulverized silex, or plaster and asbestos, upon a slide, and baked as described for full sets. The little slip of platinum kept the two teeth in place. The work shrunk somewhat; but this was remedied by again placing the piece upon the model, with the intervention of tissue paper covered with a thin coating of body. Into this I pressed the piece, till it occupied its true place, and then filled in again with more body all the crevices around the roots of the teeth, and rebaked.

After enamelling, if the work has been carefully and skillfully done upon this plan, it will be as fine a piece in appearance and fit, as can be made. It may then be soldered to a gold plate, and the little strip of platinum between the teeth be cut out. With the body and gum formerly in use, many difficulties were often encountered from discoloration of the gum, or from other injuries incurred in soldering. But with Roberts' material, these are easily avoided, and the piece can be treated the same as a block or single gum teeth. In partial sets on entire plates of platinum, I have sometimes found trouble, from the enamel giving way upon the small narrow points that connected the teeth with the plate, by the shock occasioned in biting. I have consequently left these points uncovered, and used two or three thicknesses of platinum to give greater strength. But where this is likely to occur, gold plates would be preferable, if nicely adapted with single gum teeth, or blocks of continuous gum, as the case might require. I have also applied continuous gum in cases where the natural teeth, from one to five in number, were left in the mouth, by making the plates as in full sets, cutting out around the natural ones, and raising a small bead, or placing a light wire around about one-eighth of an inch or more from the teeth, against which the gum or body is to be finished. The points around the teeth are to be left free, in order to be burnished down in case of imperfections caused by the difficulty of obtaining exact impressions in these places. In such cases, I have sometimes formed a strong standard of several thicknesses of platinum fitting closely against one or more natural teeth, leaving a loop-hole, through which to run a gold clasp for afterward securing the artificial set.

I have also secured the gold to the standard by rivets of platinum, and sometimes by two or three gold screws, not providing, in these cases, the loop-hole. These methods are to be preferred to using solder for fastening; for, in case of repair, the clasps are easily removed without leaving any foreign substances: but in case of soldering, however carefully they may be removed, there will remain some alloy, which, in the baking heat to which the piece is to be exposed, will be incorporated with the platinum. Even so small an amount of silver as may be in gold coin used for solder, will communicate a yellowish tinge to the gum, spoiling the whole work. Many operators in their early practice, I doubt not, experienced this result; and learned that no alloys, especially of silver or copper, can be admissible for soldering this work. I have tried platinum clasps without success, as no elasticity could be obtained, and therefore would not hold upon the teeth. Another source of mischief may properly be noticed in this place. In baking, especially with a new furnace, or with muffles lately renewed, either at the first or second heat, or it may be in enamelling, the piece is sometimes changed in its texture and color, as is supposed by the gases present, and the phenomenon is called gassing the piece. The body becomes porous like honey-comb, and of a bluish color. When this occurs, there is no remedy but to place it upon the metallic die, remove the whole of the injured part, and replace it with a new coating of body and gum. The teeth are seldom, if ever, thus affected. As a precaution, the muffles should be well ventilated with holes for the passage of the heated air and gases.

Though continuous gum work may at first appear tedious and beyond the reach of inexperienced dentists, they will soon find that by patience and perseverence, it may be done rapidly, and with much more satisfaction than gold work. Their patients will be better pleased with it, and the practitioners, who are influenced by professional pride, and are unwilling to place any but the best work in the mouths of their patients (except where circumstances justify the use of inferior articles), will find that they can not dispense with it.

REPAIRING.—Dentists, entirely unacquainted with the method of preparing continuous gum work, have been known to speak unfavorably of it to their patients, because of its weight, its liability to be broken, and the impossibility, in this case, of its being repaired. No substitutes for natural teeth are past finding fault with. We claim no perfection for this style; but we do insist, its defects are of less account than those of other kinds of work. It is true, the pieces are more liable to be broken by a sudden blow or fall; and for this reason, those who wear them should be especially cautioned to protect them from injury. Yet it has been proven by comparison with gold work, that the continuous gum presents fewer cases of injury, than the gold; and that these cases are by fractures resulting from carelessness in dropping the pieces. But, even if badly shattered, and several teeth should be knocked out, the piece may be soon restored, with no mark or scar indicating fracture or repair. In case of a single tooth being broken, a new one is readily inserted by entirely removing the crown of the injured one, and grinding out a niche in the gum at its base, nearly one-fourth of an inch deep, to receive the new tooth; then fill up the niche with body, and press the tooth you wish to insert down to its proper position; trim the surplus body about the neck of the tooth as in full sets, absorb the moisture with a napkin, and apply the gum to the body and wherever required.

Stay the tooth with a little plaster and asbestos placed upon its point and reaching over, so as to include the adjoining tooth on each side. A better method, however, is to place the teeth downward, imbedding their points in a paste of pulverized silex laid upon a slide, and then subject the piece to the heat required for enamelling. Once baking will generally suffice to complete this operation. But, if a piece has been more seriously injured,—say by loss of a central and canine tooth, with a point broken out from the edge of the gum, and the plate is bent so as to have lost its fit, and the gum shrunken away at any point,—we adopt a thorough method of repair. We first take an impression of the mouth and make a plaster model; upon this we place the plate, and over the point where the gum is shrunken we chip off the body and gum, and with a burnisher work the plate down wherever required, to exactly fit the model. We then make a niche for each tooth to be inserted, apply the body and gum, stay the teeth, and bake the piece as above described. If it has been thoroughly packed, two bakings will be sufficient, but sometimes three may be required. The only rule is, to repeat the operation till the object is accomplished, taking care always not to overheat the piece. In case of the piece having been long worn, it is well, before doing any thing to it, to subject it to a moderate degree of heat, sufficient to burn off any impurities it may have collected in the mouth.

Another set may be presented for repair, literally broken to pieces; but the plate remaining a perfect fit, and some of the teeth being undisturbed, we wish to avail ourselves of these. We therefore make a dam of putty around the edge of the plate, and run in fusible metal to form a cast, which shall serve as a support for the plate. Not to endanger cracking the teeth by the heat, the piece may be placed in a dish containing a little water, and after the metal is poured and begins to harden, more water may be added, so as to cover the whole mass. But this is hardly necessary, if the alloy be run nearly at its cooling temperature.

The piece being now supported upon the cast, and held firmly with one hand, a small chisel, made from an excavator, may be applied with the other to the old material which it is desirable to remove, and an assistant gently tapping this with a hammer, the body is quickly chipped off between the teeth, and wherever the chisel is directed without injury to other parts, or without mis-shaping the plate. The teeth may now be inserted and soldered to the plate, with the old or new linings if required, and the new body and gum may then be applied, as in making a new piece. All this may seem to involve much time and trouble; but little more is really required than to repair a gold plate. The cast of fusible metal is much more readily made than a plaster cast. The old body and gum is removed and new applied, in shorter time than two gum teeth can be fitted upon the gold plate, and the soldering and first baking of the body requires no more time than to solder and cool the gold work. We then have, in the one case, the gum to be applied, and in the other, the gold is to be polished; so that the whole difference of time involved, however great the repairs, need not be more than one or two hours. But a single tooth may be repaired as soon as a gum tooth on gold plate. One difficulty, often met with in mending, requires to be noticed. It arises from the necessity of employing mixtures likely to be of different composition from that of the original body; and possibly more infusible. In this case, the old body will melt, before the new has bcome incorporated with it, thus ruining the work by overheating. In some instances, after one baking, I have been obliged to condemn the whole work and entirely replace the old body. This difficulty is now obviated by being able to use always the same material; it being prepared in large quantities, by Dr. E. A. L. Roberts (to the amount of a ton weight at a time), so that the supply of uniform composition can be depended upon for years to come.

CONCLUDING REMARKS.—In looking over this paper, which has already reached a length I did not anticipate, it is easy to perceive that numerous points have escaped notice, which, to the experienced manipulator, are of trivial importance; but to the student, without more detailed instruction, may be insurmountable obstacles to his farther progress. If, however, the latter possess a fair endowment of skill and ingenuity, he need not despair of success.

The plain outline of the various operations, as given above, may be his general guide which will lead him to good results; but his own judgment, skill and taste, moreover, must be repeatedly exercised, independently of my instructions, however minutely they may be given. Like all other processes, this can not claim to have reached perfection, and, therefore, there is still room for improvement. It admits of infinite variety in its application, and hence presents a fine field for artistic taste and invention; which, I can not doubt, will be well improved by the genius and energy exhibited by the Dental profession in all such directions. There is a fascination about this style of work, which no other possesses, as it seems to compete, in point of beauty, with nature itself.

